# Domain Adaptation Based on Semi-Supervised Cross-Domain Mean Discriminative Analysis and Kernel Transfer Extreme Learning Machine

**DOI:** 10.3390/s23136102

**Published:** 2023-07-02

**Authors:** Xinghai Li, Jianwei Ma

**Affiliations:** College of Information Engineering, Henan University of Science and Technology, Kaiyuan Avenue, Luoyang 471023, China; li_xinghai@163.com

**Keywords:** cross-domain mean approximation, semi-supervised discriminant analysis, kernel extreme learning machine, domain adaptation

## Abstract

Good data feature representation and high precision classifiers are the key steps for pattern recognition. However, when the data distributions between testing samples and training samples do not match, the traditional feature extraction methods and classification models usually degrade. In this paper, we propose a domain adaptation approach to handle this problem. In our method, we first introduce cross-domain mean approximation (CDMA) into semi-supervised discriminative analysis (SDA) and design semi-supervised cross-domain mean discriminative analysis (SCDMDA) to extract shared features across domains. Secondly, a kernel extreme learning machine (KELM) is applied as a subsequent classifier for the classification task. Moreover, we design a cross-domain mean constraint term on the source domain into KELM and construct a kernel transfer extreme learning machine (KTELM) to further promote knowledge transfer. Finally, the experimental results from four real-world cross-domain visual datasets prove that the proposed method is more competitive than many other state-of-the-art methods.

## 1. Introduction

Traditional classification tasks deal with situations where the distribution of source domain samples and target domain samples are the same, and a classifier trained from source domain samples can be directly applied to the target domain samples. Theoretical studies on classifiers are also based on this assumption [1]. However, in real-world environments, the distribution of source domain samples and target domain samples is often different due to factors such as lighting, viewpoints, weather conditions, and cameras [2]. The most advanced classifiers trained on source domain samples may dramatically degrade when applied to target domain samples. One possible solution is to annotate the new data and retrain the model. Unfortunately, labeling a large number of samples is costly and time-consuming. Another promising solution is domain adaptation (DA), which aims to minimize the distribution gap between the two domains and learn a fairly accurate shared classifier for both domains, where the target domain samples do not have available labels.

One strategy of DA is to find invariant feature representation subspaces between domains to minimize distribution divergence. A large number of existing methods applying this strategy learn a shared feature subspace where the distribution mismatch between the two domains is reduced, and then employ standard classification methods in this subspace [3]. Metrics that measure the mismatch in distribution between domains include maximum mean discrepancy (MMD) [4], Kullback–Leibler (KL) divergence [5], Bregman divergence [6], and Wasserstein distance [7], among others. Due to the fact that MMD is a nonparametric estimation criterion for distance, researchers have proposed various adaptive models commonly with this metric. Pan et al. [8] proposed a classical DA method maximum mean difference embedding (MMDE) by defining a semidefinite program (SDP) and introducing the MMD criterion to match marginal distribution. To further reduce the computational burden of the MMDE and to preserve the key features of the data, [9] proposed an improved method, transfer component analysis (TCA), which reduces the differences in marginal distribution between domains by projecting the data into a latent feature space. Gong et al. [10] developed geodesic flow kernel (GFK), which is another classical method. GFK establishes connections between domains based on a series of intermediate subspaces instead of using only one subspace. In addition to marginal distributions, the joint distribution adaptive (JDA) [11] approach also considers conditional distribution matching, where target samples are iteratively assigned pseudo-labels. Based on JDA, various variants have been developed. Lu et al. [12] introduced weight adaptation into JDA, paid attention to the feature representation of a single sample, emphasized the difference between samples in the target domain learning, and proposed the weights adaptation joint distribution adaptation (W-JDA). A joint distribution adaptation based transfer network with diverse feature aggregation (JDFA) is proposed by Jia et al. [13], which enhances feature extraction capability across large domain gaps through the diverse feature aggregation module; then, MMD was employed to reduce distribution differences.

Huang et al. [14] proposed the extreme learning machine (ELM) model, which is a “generalized” single-hidden-layer feedforward neural network that has been employed to address classification and regression problems. The hidden layer nodes of the ELM are randomly initialized and without tuning, and the output weights between the hidden and output layers can be analytically determined [15]. Many researchers have extended it in theory and application, and proposed numerous new ELMs, such as AdaBoost ELM [16], kernel-based multilayer ELM (ML-KELM) [17], voting-based ELM (V-ELM) [18], Kalman filter ELM (KA-ELM) [19], and others. However, the above algorithms require a prerequisite assumption that there is consistency in the distribution of labeled training samples and testing samples, and therefore these classical ELM algorithms cannot handle domain adaptation problem. To address this issue, some improvements have been made to ELM. Zang et al. [20] proposed a two-stage transfer limit learning machine (TSTELM) framework. The framework uses MMD to reduce the distribution differences of output layers between domains in the statistical matching stage. In the subspace alignment stage, the source and target model parameters are aligned, and output weight approximation is added to further promote knowledge transfer across domains. Chen et al. [21] developed a DST-ELM method to learn a new feature space. With the target domain discrimination information fixed in the feature space, the source domain samples are adapted to the target domain and the MMD distance between them is minimized. The MMD metric has been used in the above methods to measure the distribution differences between the source domain samples and the target domain samples as a whole, but they ignore the impact of individual samples on the distribution differences.

To sum up, there are several challenges to DA. The first issue is the similarity between source domain and target domain. To cope with this issue, suitable distribution distance measurement criteria are found to eliminate this dissimilarity. Secondly, there is class separability and shared feature extraction between source and target domains in the feature subspace. The third challenge is the knowledge transfer ability of the classifier.

**Figure 1:**
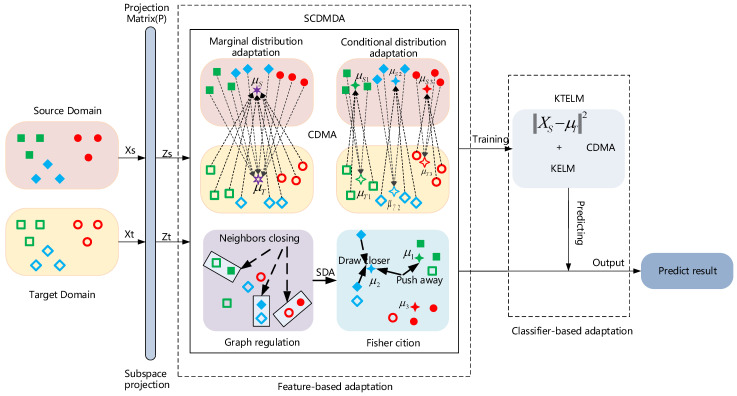
Samples of the same color are of the same class in the source and target domains. An illustration of our method: (1) SCDMDA is designed by introducing CDMA into SDA for shared feature extraction across domains. (2) KTELM is constructed by adding a cross-domain mean approximation constraint into a kernel extreme learning machine for classification. (3) SCDMDA and KTELM are combined for cross-domain feature extraction and classification.

We believe that a well-developed DA algorithm is able to address these three challenges. Therefore, we want to tackle all these problems simultaneously. We propose a domain adaptation approach which consists of two stages: feature-based adaptation and classifier-based adaptation. At the former stage, we develop semi-supervised cross-domain mean discriminative analysis (SCDMDA). It introduces cross-domain mean approximation (CDMA) [17] into SDA to minimize the marginal and conditional interdomain distribution discrepancy. Meanwhile, the category separability of LDA and the original structure information of graph regularizers are used together to capture shared features between the source and target domains. At the latter stage, we first choose KELM as the classification model. Then, a kernel transfer extreme learning machine (KTELM) is designed by adding a cross-domain mean constraint term into KELM to further enhance the knowledge transfer ability of our method, as shown in Figure 1. Finally, we investigate the performance of our approach on public domain adaptation datasets, and the experimental results show that it can learn better domain-invariant features with high accuracy and outperform other existing shallow and deep domain adaptation methods.

In this paper, we make the following contributions:

- We introduce CDMA into SDA and then propose SCDMDA. It extracts shared discriminative features across domains by using CDMA to minimize the marginal and conditional discrepancies between domains and applying SDA to exploit the label information and original structure information.
- We present KTELM by designing a cross-domain mean approximation constraint into KELM for classification in domain adaptation.
- We obtain a classifier with the ability of knowledge transfer by combining SCDMDA and KTELM and implement a classification task on public image datasets. The results show the superiority of our approach.

The rest of this paper is as follows: In Section 2, we briefly describe SDA, ELM, KELM, and DA. Section 3 provides SCDMDA and KTELM algorithms. The experimental results and analysis are presented in Section 4. Finally, Section 5 is the conclusion of this paper.

## 2. Preliminary

In this section, we will briefly introduce SDA, ELM, KELM, and DA.

### 2.1. Semi-Supervised Discriminant Analysis (SDA)

As a classical semi-supervised feature extraction algorithm, SDA extends LDA by adding a geometrical regularizer to prevent overfitting. Given a sample set $X=X_{l}\cup X_{u}={\left\{\left(x_{r},y_{r}\right)\right\}}_{r=1}^{n_{l}}\cup {\left\{x_{v}\right\}}_{v=1}^{n_{u}}\in R^{d\times N}$, it is divided into two parts: a labeled subset $X_{l}$ and an unlabeled subset $X_{u}$. $x_{r}$ is a labeled sample with a label $y_{r}$, and $x_{v}$ is an unlabeled sample. $n_{l}$ and $n_{u}$ are the number of labeled and unlabeled samples, respectively, $N=n_{l}+n_{u}$. The dimension of the sample is d. We can define the objective function of SDA as follows:

(1) $$J_{1}(P)=\arg\;\max\frac{P^{T}S_{b}P}{P^{T}S_{w}P+\alpha P^{T}XQX^{T}P}$$

where P is the projection matrix; Q=D−G is Laplacian matrix. G is the graph neighbor similarity matrix of X. $G_{ij}=\left\{\begin{array}{l} 1\;,\;if\;x_{i}\in N_{p}(x_{j})\;or\;x_{j}\in N_{p}(x_{i})\\ 0\;,\;otherwise \end{array}\right.$, where $x_{i}$ and $x_{j}$ are among p nearest neighbors of each other, and $N_{p}(x_{i})$ denotes the set of p nearest neighbors of $x_{i}$. D is a diagonal matrix; its entries are a column (or a row, since G is symmetric) sum of G, $D_{ii}=\sum_{j}G_{ij}$. α is the parameter balancing the graph regularization. $S_{b}$ and $S_{w}$ represent the interclass scatter matrix and between-class scatter matrix, respectively, which are calculated as follows:

(2) $$S_{b}=\sum_{c=1}^{C}N_{c}(m^{\left(c\right)}-u){\left(m^{\left(c\right)}-u\right)}^{T}$$

(3) $$S_{w}=\sum_{c=1}^{C}\sum_{i=1}^{N_{c}}(x_{i}^{\left(c\right)}-m^{\left(c\right)}){\left(x_{i}^{\left(c\right)}-m^{\left(c\right)}\right)}^{T}$$

where $u=\frac{1}{N}\sum_{i=1}^{N}x_{i}$, and $m^{\left(c\right)}=\frac{1}{N_{c}}\sum_{i=1}^{N_{c}}x_{i}^{\left(c\right)}$. C is the number of class, $x_{i}^{\left(c\right)}$ is the i-th sample belonging to class c, $N_{c}$ represents the number $x_{i}^{\left(c\right)}$, and $m^{\left(c\right)}$ is the mean value of samples of class c.

### 2.2. Extreme Learning Machine (ELM) and Kernel Extreme Learning Machine (KELM)

As a high-performance classifier, ELM with a single hidden layer has a fast training process and high classification performance. Given a dataset ${\left\{\left(x_{i},y_{i}\right)\right\}}_{i=1}^{N}$ with N samples $x_{i}$ and its label $y_{i}$, we could construct an ELM network with L hidden nodes as follows:

(4) $$y_{i}^{T}=\sum_{j=1}^{L}W_{j}^{T}g(w_{j}x_{i}+b_{j})$$

where $y_{i}$ is the output prediction result of sample $x_{i}$, and $W_{j}$ is the output weight connecting the hidden layer and output layer. g(x) is an activation function to improve the nonlinear fitting ability of the network. $w_{j}$ and $b_{j}$ are input weight and bias, between the input layer and the hidden layer, which can be set randomly. It can be seen that $W_{j}$ is the key parameter of an ELM network. If we want to obtain an optimal result, the following objective function needs to be solved:

(5) $$J_{2}(W)\;=\underset{W}{\min}\frac{1}{2}{\left\|W\right\|}^{2}+\frac{\lambda }{2}{\left\|Y-HW\right\|}^{2}$$

In Equation (5), $H={\left[g{\left(x_{i}\right)}^{T},\cdots ,g{\left(x_{N}\right)}^{T}\right]}^{T}$, $W={\left[W_{1},\cdots ,W_{L}\right]}^{T}$, and $Y={\left[y_{1},\cdots ,y_{N}\right]}^{T}$. ${\left\|W\right\|}^{2}$ is a regularizer to avoid model overfitting, and ${\left\|Y-HW\right\|}^{2}$ is the training error. λ is a tradeoff parameter between minimizing training errors and the regularizer.

We consider Equation (5) to be a regular least square problem and set $\frac{\partial J_{2}}{\partial W}=0$. The optimal solution of W is calculated as follows:

(6) $$W^{*}=\left\{\begin{array}{cc} {\left(H^{T}H+\frac{I_{L}}{\lambda }\right)}^{-1}H^{T}Y, & N>L\\ H^{T}{\left(H^{T}H+\frac{I_{N}}{\lambda }\right)}^{-1}Y, & N\leq L \end{array}\right.$$

Then, the output of the trained ELM model corresponding to test sample $x_{Te}$ is

(7) $$y_{Te}=\left\{\begin{array}{c} \;\mathrm{sign}(h_{{}^{Te}}^{T}W^{*})\\ \arg\max(h_{{}^{Te}}^{T}W^{*}) \end{array}\right.\begin{array}{c} ,\;for\;binary\;classification\\ ,\;for\;multi-classification \end{array}$$

where $h_{Te}=g(x_{Te})$.

If the activation function g(x) is unknown, we let $K=HH^{T}$, $K_{\left(ij\right)}=g(x_{i})g{\left(x_{j}\right)}^{T}=\Omega (x_{i},x_{j})$, $\Omega (x_{i},x_{j})$ is a kernel function. The output of KELM can be defined as:

(8) $$y_{Te}=\left\{\begin{array}{c} \;\mathrm{sign}\left(g\left(x_{Te}\right)H^{T}{\left(K+\frac{I_{L}}{\lambda }\right)}^{-1}Y\right)\;,\;for\;binary\;classification\\ \arg\max\left(g\left(x_{Te}\right)H^{T}{\left(K+\frac{I_{N}}{\lambda }\right)}^{-1}Y\right),for\;multi-classification \end{array}\right.$$

where $g\left(x_{Te}\right)H^{T}=\left[\begin{array}{c} \Omega (x,x_{1})\\ \vdots \\ \Omega (x,x_{N}) \end{array}\right]$.

### 2.3. Domain Adaptation (DA)

In practical application scenarios, it is difficult to collect enough training samples with the same distribution as the testing samples. For example, in photography, it is hard to avoid changes in pixel distribution caused by changes in lighting, posture, weather, and background. If a large number of samples with the same distribution are selected and manually marked for model training, the cost will be high [22]. The above distribution changes will generate a domain bias between the training dataset and the testing dataset, which could degrade the performance of traditional machine learning methods. DA can not only apply knowledge from other domains (source domains) that contain substantial labeled samples and are related to the target domain for model training, but also eliminate domain bias. Existing DA methods are mainly divided into instance-based adaptation, feature-based adaptation, and classifier-based adaptation.

The instance-based adaptation methods seek a strategy which selects “good” samples in the source domain to participate in model training and suppresses “bad” samples to prevent negative transfer. Kernel mean matching (KMM) [23] minimizes the maximum mean difference [24] and weighs the source data and target data in the reproducing kernel Hilbert space (RKHS), which can correct the inconsistent distribution between domains. As a classic instance-based adaptation method, transfer adaptive boosting (TrAdaBoost) [25] extends the AdaBoost algorithm to weigh source-labeled samples and target-labeled samples to match the distributions between domains. In the work [26], a simple two-stage algorithm was proposed to reweight the results of testing data from the training classifier using their signed distance to the domain separator. Moreover, it applied manifold regularization to propagate the labels of target instances with larger weights to those with smaller weights. Instance-based adaptation methods are more efficient for knowledge transfer, but negative transfer [27] can easily occur when there are no shared samples between domains.

The feature-based adaptation methods attempt to learn a subspace with a better shared features representation, in which the marginal or conditional distribution divergences between domains are minimized to facilitate knowledge transfer. Transfer component analysis (TCA), joint distribution adaptation (JDA), balanced distribution adaptation (BDA), and multiple kernel variant of maximum mean discrepancy (MK-MMD) apply the maximum mean discrepancy (MMD) strategy to perform distribution matching, thus minimizing the marginal or conditional distribution divergences between domains [28]. In [29], Wang et al. use the locality preserving projection (LPP) to learn a joint subspace from the source and target domains. 

In recent years, deep neural networks (DNNs) have performed well in high-level semantic feature extraction with strong expression ability, and have broad application prospects in domain adaptation. Domain adaptation networks (DAN) [30], faster domain adaptation networks (FDAN) [31], residual transfer networks (RTN) [32], domain adversarial neural networks (DANN) [33], and collaborative and adversarial networks (CAN) [34] use MMD to align distribution discrepancies in an end-to-end learning paradigm for feature extraction across domains. However, DNNs also have their shortcomings, for example, the models contain huge parameters and require a large amount of data for training, which is not suitable for small sample data. DNNs have a strong ability to fit data and can approximate any complex objective function, but the computational cost is high. Our method in this paper has a small number of parameters and fast calculation speed, and it is suitable for small sample data classification.

The goal of the classifier-based adaptation methods is to adjust the parameters of the classifier so that the classifier trained on the source domain has good performance in the target task. Adaptive support vector machine (Adapt-SVM) [35] introduced a regularizer into SVM to minimize the classification error and the discrepancy between two classifiers trained on the source- and target-labeled samples. Based on LS-SVM, Tommasi et al. [36] presented an adaptation model called multi-model knowledge transfer (Multi-KT), in which the objective function imposed the requirement that the target classifier and a linear combination of the source classifiers be close to each other for knowledge transfer. Considering the simplicity and efficiency of ELM, many adaptation models based on ELM have been proposed for domain adaptation, such as joint domain adaptation semi-supervised extreme learning machine (JDA-S2ELM) [37], cross-domain ELM (CDELM) [38], domain space transfer ELM (DST-ELM) [21], domain adaptation extreme learning machine (DAELM) [39], and so on.

In addition, the adaptation model based on the generative adversarial network (adversarial-based adaptation) [34,40,41,42] has recently shown exceptional performance. Many target samples with transferable and domain-invariant features are produced through the generator of adversarial learning and then applied to confuse the domain discriminator training on the source domain and optimize the generator. It can effectively reduce the distribution differences between domains and transfer knowledge efficiently. Since generating samples with domain-invariant features is the main task of adversarial-based adaptation, it is commonly attributed to feature-based adaptation.

In this paper, our method is a combination of feature- and classifier-based adaptation. SCDMDA belongs to a shallow feature-based adaptation method, and KTELM is a classifier-based adaptation method. At the feature-based adaptation stage, SCDMDA finds domain-invariant features. It not only applies SDA to maximize between-class scatter, minimize within-class scatter, and keep the original structure information by pseudo-labeled target samples and labeled source samples, but it also reduces the distribution differences between domains using CDMA. At the classifier-based adaptation stage, a cross-domain mean constraint term is added to KELM to further enhance its knowledge transfer ability.

## 3. Proposed Method

In a domain adaptation environment, given a source domain with labeled samples $X_{S}={\left\{\left(x_{i},y_{i}\right)\right\}}_{i=1}^{n_{S}}\in R^{d\times n_{S}}$ and a target domain with unlabeled samples $X_{T}={\left\{x_{j}\right\}}_{j=1}^{n_{T}}\in R^{d\times n_{T}}$, where the distributions between $X_{S}$ and $X_{T}$ are not the same but are related and the label space $Y={\left\{y_{c}\right\}}_{c=1}^{C}$ is shared, the number of categories is denoted by C. To address the domain adaptation problem, our proposed method is divided into two stages: feature-based adaptation and classifier-based adaptation.

At the feature-based adaptation stage, we design SCDMDA, and its goal is to find an optimal transformation matrix $P=(p_{1},p_{2},\cdots ,p_{k})\in R^{d\times k}$, which embeds $X_{S}$ and $X_{T}$ into low-dimensional subspaces $z_{S}=P^{T}x_{S}\in R^{k}$ and $z_{T}=P^{T}x_{T}\in R^{k}$, where k is the subspace dimension. In this subspace, the distributions between $Z_{S}$ and $Z_{T}$ are closer than those between $X_{S}$ and $X_{T}$.

At the classifier-based adaptation stage, KTELM is constructed by adding a cross-domain mean constraint term into KELM, which improves the knowledge transfer ability of KELM.

### 3.1. Feature-Based Adaptation of SCDMDA

#### 3.1.1. Cross-Domain Mean Approximation (CDMA)

From Equation (1) in Section 2.1, we can see that although SDA has better feature extraction performance than LDA, it can obtain bad feature representation when the training samples and testing samples have inconsistent data distributions. To address this issue, we need to reduce the distribution discrepancy of training samples and testing samples with the help of domain adaptation technology based on CDMA. It is expressed as follows:

(9) $$\begin{array}{ll} L_{CDMA}\left(P\right) & =\underset{P}{\min}\left(\sum_{i=1}^{n_{S}+n_{T}}{\left\|Px_{i}-P\mu \right\|}_{2}^{2}+\sum_{c=1}^{C}\sum_{i=1}^{n_{Si}^{\left(c\right)}+n_{Ti}^{\left(c\right)}}{\left\|Px_{i}^{\left(c\right)}-P\mu^{\left(c\right)}\right\|}_{2}^{2}\right)\\ & =\underset{P}{\min}\mathrm{tr}\left(P^{T}M_{0}P+P^{T}M_{c}P\right)\\ s.t. & P^{T}X^{T}GXP=I \end{array}$$

where $\mu =\left\{\begin{array}{c} \mu_{T},\;\;\;\;\;\mathrm{if}\;\;\;x_{i}\in X_{S}\\ \mu_{S},\;\;\;\;\;\mathrm{if}\;\;\;x_{i}\in X_{T} \end{array}\right.$ and $\mu^{\left(c\right)}=\left\{\begin{array}{c} \mu_{T}^{\left(c\right)},\;\;\;\;\;\mathrm{if}\;\;\;x_{i}^{\left(c\right)}\in X_{S}^{\left(c\right)}\\ \mu_{S}^{\left(c\right)},\;\;\;\;\;\mathrm{if}\;\;\;x_{i}^{\left(c\right)}\in X_{T}^{\left(c\right)} \end{array}\right.$. $\mu_{T\left(S\right)}$ is the mean vector of the target (source) domain samples, and $\mu_{T\left(S\right)}^{\left(c\right)}$ is the mean vector of the target (source) domain $X_{T\left(S\right)}^{\left(c\right)}$ with c category. $n_{Si}^{\left(c\right)}$ and $n_{Ti}^{\left(c\right)}$ are the number of samples in $X_{S}^{\left(c\right)}$ and $X_{T}^{\left(c\right)}$. $M_{0}=X^{T}X-X^{T}\mu -X\mu^{T}+\mu^{T}\mu$, $M_{c}=\sum_{c=1}^{C}\;(X^{\left(c\right)}{}^{T}(X^{\left(c\right)})-{\left(X^{\left(c\right)}\right)}^{T}\mu^{\left(c\right)}-X^{\left(c\right)}{\left(\mu^{\left(c\right)}\right)}^{T}+\mu^{\left(c\right)}{}^{T}(\mu^{\left(c\right)}))$, $X=\left(\begin{array}{c} X_{S}\\ X_{T} \end{array}\right)$, and $X^{\left(c\right)}=\left(\begin{array}{c} X_{S}^{\left(c\right)}\\ X_{T}^{\left(c\right)} \end{array}\right)$. $G=I-\frac{1}{n}qq^{T}$ is the center matrix, where $q\in R^{n}$ is the column vector with element 1, $n=n_{S}+n_{T}$. In Equation (9), CDMA adapts both the marginal distribution in the first term and the conditional distribution in the second term.

#### 3.1.2. Semi-supervised Cross-Domain Mean Discriminative Analysis (SCDMDA)

To enhance the knowledge transfer ability of SDA, we combine Equation (9) and Equation (1) and design the objective function of SCDMDA for shared feature extraction across domains as follows:

(10) $$\begin{array}{l} J_{3}(P)=\underset{P}{\min}\;\mathrm{tr}\left(\frac{P^{T}\left(\beta S_{w}+\alpha XQX^{T}\right)P}{\beta P^{T}S_{b}P}+P^{T}X\left(M_{0}+M_{c}\right)X^{T}P\right)+\lambda_{1}{\left\|P\right\|}_{F}^{2}\\ s.t.\;\;\;\;\;\;\;\;\;P^{T}X^{T}GXP=I \end{array}$$

Furthermore, the above formula is equivalent to:

(11) $$\begin{array}{l} J_{3}(P)=\underset{P}{\min}\;\mathrm{tr}P^{T}\left(X^{T}\left(M_{0}+M_{c}\right)X+\alpha X^{T}QX+\beta \left(S_{w}-S_{b}\right)\right)P+\lambda_{1}{\left\|P\right\|}_{F}^{2}\\ s.t.\;\;\;\;\;\;\;\;\;\;\;\;\;\;P^{T}X^{T}GXP=I \end{array}$$

where ${\left\|P\right\|}_{F}^{2}$ with tradeoff parameter $\lambda_{1}$ is introduced into the objective function to ensure the sparsity of matrix P. α and β balance the influence of the graph regularizer and LDA, respectively.

We set $\frac{\partial J_{3}}{\partial P}=0$, and Equation (11) becomes a generalized eigen-decomposition problem:

(12) $$\left(X^{T}\left(\left(M_{0}+M_{c}\right)+\alpha Q\right)X+\beta \left(S_{w}-S_{b}\right)+\lambda_{1}Ι\right)P=GP\Phi$$

Finally, Equation (12) is used to find k, the smallest eigenvectors for the optimal adaptation matrix P, to reduce the difference distribution between domains.

To address the issue of the nonlinear data, we transform Equations (11) and (12) into a high-dimensional space by kernel mapping, namely $x\overset{mapping}{\to }\varphi (x)$. The kernel matrix corresponding to the dataset X is $K=\varphi^{T}(X)\varphi (X)$, then we obtain:

(13) $$\begin{array}{l} J_{3}(P)=\underset{P}{\min}\;\mathrm{tr}P^{T}\left(K^{T}\left(M_{0}+M_{c}\right)K+\alpha K^{T}QK+\beta \left(S_{w}-S_{b}\right)\right)P+\lambda_{1}{\left\|P\right\|}_{F}^{2}\\ s.t.\;\;\;\;\;\;\;\;\;\;\;\;\;\;P^{T}K^{T}GKP=I \end{array}$$

(14) $$\left(K^{T}\left(\left(M_{0}+M_{c}\right)+\alpha Q\right)K+\beta \left(S_{w}-S_{b}\right)+\lambda_{1}Ι\right)P=GP\Phi$$

where $S_{w}^{\varphi }$ and $S_{b}^{\varphi }$ are the nonlinear forms of $S_{w}$ and $S_{b}$ under the kernel map, respectively, which can be obtained through Ref. [43]. A complete procedure of SCDMDA is summarized in Algorithm 1.
**Algorithm 1**. SCDMDA**Input**: Dataset $X_{S}$ and $X_{T}$, subspace dimension k, parameters α, β, $\lambda_{1}$, θ, and $\lambda_{2}$, classifier KTELM, maximum number of iterations $T_{\max}$.
**Output**: Projection matrix P and target prediction ${\tilde{Y}}_{T}$. Step1: According to Equations (1) and (9), construct Q, G, and $M_{0}$, and set $S_{w}$ and $S_{b}$ to 0. Step2: Let t=1. Step3: Solve Equation (12) or Equation (14) to obtain the projection matrix P. Step4: Project $X_{S}$ and $X_{T}$ by P into k-dimensional subspace to obtain $Z_{S}$ and $Z_{T}$. Step5: Learn a KTELM on $\left\{Z_{S},Y_{S}\right\}$, and classify $Z_{T}$ to obtain the label set of the target domain data ${\tilde{Y}}_{T}$. Step6: Use $\left\{X_{S},Y_{S}\right\}$ and $\left\{X_{T},{\tilde{Y}}_{T}\right\}$, construct $M_{c}$, and solve $S_{w}$ and $S_{b}$ ($S_{w}^{\varphi }$ and $S_{b}^{\varphi }$ in the nonlinear case) according to Equations (2) and (3). Step7: Let t←t+1. Step8: If $t\geq T_{\max}$ or ${\tilde{Y}}_{T}$ does not change, output P, otherwise, go to Step3.

### 3.2. Classifier-Based Adaptation of KTELM

After feature adaptation of SCDMADA, we can obtain P and obtain $Z_{S}=P^{T}X_{S}$ and $Z_{T}=P^{T}X_{T}$. Then, it can be seen from Section 2.2 that we can train a KELM on $\left\{Z_{S},Y_{S}\right\}$ and effectively predict $Z_{T}$. However, we can see from Equation (5) that KELM has no capacity for knowledge transfer. In this section, we design a cross-domain mean constraint on the source domain as follows:

(15) $$J_{4}(W)=\underset{W}{\min}{\left\|H_{S}W-H_{\mathrm{Tav}}W\right\|}^{2}+\sum_{c=1}^{C}\left({\left\|H_{S}^{\left(c\right)}W-H_{\mathrm{Tav}}^{\left(c\right)}W\right\|}^{2}\right)$$

where $H_{S}^{\left(c\right)}$ is the samples dataset with c category in $H_{S}$, and $H_{\mathrm{Tav}}^{\left(c\right)}$ denotes the mean of the samples dataset with c category in $H_{T}$. Then, we add Equation (15) into Equation (5) and obtain

(16) $$J_{5}(W)\;=\underset{W}{\min}\frac{1}{2}{\left\|W\right\|}^{2}+\frac{\lambda_{2}}{2}{\left\|Y_{S}-H_{S}W\right\|}^{2}+\theta \left({\left\|H_{S}W-H_{\mathrm{Tav}}W\right\|}^{2}+\sum_{c=1}^{C}\left({\left\|H_{S}^{\left(c\right)}W-H_{\mathrm{Tav}}^{\left(c\right)}W\right\|}^{2}\right)\right)$$

where $\lambda_{2}$ and θ balance the influence of training errors and cross-domain mean constraint on the source domain, respectively.

Similar to ELM, we set $\frac{\partial J_{5}}{\partial W}=0$ and obtain

(17) $$W^{*}=H_{S}^{T}\left(H_{S}^{T}H_{S}+\theta \left(\begin{array}{c} \left(H_{S}-H_{\mathrm{Tav}}\right){\left(H_{S}-H_{\mathrm{Tav}}\right)}^{T}\\ +\sum_{c=1}^{C}\left(H_{S}^{\left(c\right)}-H_{\mathrm{Tav}}^{\left(c\right)}\right){\left(H_{S}^{\left(c\right)}-H_{\mathrm{Tav}}^{\left(c\right)}\right)}^{T} \end{array}\right)+\frac{I_{N}}{\lambda_{2}}\right)$$

We kernelize Equation (17) and let $K_{S}=H_{S}^{T}H_{S}$, $K_{S(ij)}=g(x_{Si})g{\left(x_{Sj}\right)}^{T}=\Omega (x_{Si},x_{Sj})$, $\Omega (x_{i},x_{j})$ is a kernel function. The output of KTELM can be defined as:

(18) $$y_{T}=\left\{\begin{array}{c} \;\mathrm{sign}\left(g\left(x_{T}\right)H^{T}{\left(K_{S}+\theta \left(\begin{array}{c} {\left(\varphi_{S}-\varphi_{\mathrm{Tav}}\right)}^{T}\left(\varphi_{S}-\varphi_{\mathrm{Tav}}\right)\\ +\sum_{c=1}^{C}{\left(\varphi_{S}^{\left(c\right)}-\varphi_{\mathrm{Tav}}^{\left(c\right)}\right)}^{T}\left(\varphi_{S}^{\left(c\right)}-\varphi_{\mathrm{Tav}}^{\left(c\right)}\right) \end{array}\right)+\frac{I_{L}}{\lambda_{2}}\right)}^{-1}Y\right)\;,\;for\;binary\;classification\\ \arg\max\left(g\left(x_{T}\right)H^{T}{\left(K_{S}+\theta \left(\begin{array}{c} {\left(\varphi_{S}-\varphi_{\mathrm{Tav}}\right)}^{T}\left(\varphi_{S}-\varphi_{\mathrm{Tav}}\right)\\ +\sum_{c=1}^{C}{\left(\varphi_{S}^{\left(c\right)}-\varphi_{\mathrm{Tav}}^{\left(c\right)}\right)}^{T}\left(\varphi_{S}^{\left(c\right)}-\varphi_{\mathrm{Tav}}^{\left(c\right)}\right) \end{array}\right)+\frac{I_{N}}{\lambda_{2}}\right)}^{-1}Y\right)\;,\;for\;multi-classification \end{array}\right.$$

where $g\left(x_{T}\right)H^{T}=\left[\begin{array}{c} \Omega (x_{Tj},x_{1})\\ \vdots \\ \Omega (x_{Tj},x_{n_{T}}) \end{array}\right]$, $\varphi_{S}=\left[\begin{array}{c} \Omega (x_{Si},x_{1})\\ \vdots \\ \Omega (x_{Si},x_{n_{S}}) \end{array}\right]$, and $\varphi_{\mathrm{Tav}}$ is the mean of $\left[\begin{array}{c} \Omega (x_{Tj},x_{1})\\ \vdots \\ \Omega (x_{Tj},x_{n_{T}}) \end{array}\right]$. $\varphi_{S}^{\left(c\right)}=\left[\begin{array}{c} \Omega (x_{Si}^{\left(c\right)},x_{1}^{\left(c\right)})\\ \vdots \\ \Omega (x_{Si}^{\left(c\right)},x_{n_{S}}^{\left(c\right)}) \end{array}\right]$, and $\varphi_{\mathrm{Tav}}^{\left(c\right)}$ is the mean of $\left[\begin{array}{c} \Omega (x_{Tj}^{\left(c\right)},x_{1}^{\left(c\right)})\\ \vdots \\ \Omega (x_{Tj}^{\left(c\right)},x_{n_{T}}^{\left(c\right)}) \end{array}\right]$.

### 3.3. Discussion

In this paper, our method is proposed for domain adaptation, which includes feature adaptation based on SCDMDA and classifier adaptation based on KTELM.

- From Equation (11) and Equation (18), it can be seen that, compared with SDA, SCDMDA adopts CDMA to reduce the distribution discrepancy between domains, which is better for domain adaptation than SDA. Moreover, as a semi-supervised feature extraction method, SCDMDA focuses more attention on individual information with the help of the category separability of LDA and the original structure information of graph regularizers.
- Compared with MMD, CDMA reflects individual differences. In our method, CDMA mines individual information through $M_{0}$ and $M_{c}$, which is a more effective interdomain distribution difference measurement mechanism than MMD. In addition, we verify the improved method on k-NN, KELM, and KTELM classifiers.
- In the classical ELM, solving the output weight W that connects the hidden layer and the output layer is the key, and the optimal solution is obtained by solving Equation (5). However, for samples with interdomain distribution differences, the solution obtained by Equation (5) is not the optimal. The domain adaptation is added to the ELM to obtain Equation (16), and the optimal $W^{*}$ can be obtained. By adopting the cross-domain mean constraint on the source domain to achieve the cross-domain transfer of knowledge, the interdomain distribution difference can be reduced, which shows that KTELM has higher domain adaptation accuracy.

## 4. Experiments and Analysis

In this section, extensive experiments were conducted on four widely adopted datasets, including Office+Caltech [10], Office-31 [44] object recognition, USPS [45] and MNIST [46] digital handwriting, and PIE face [47], to test SCDMDA+KETLM. Table 1 shows some descriptions of these. All the experiments were conducted on a PC equipped with Win10, an Intel i5 10400F, 2.9 GHz CPU, and 8 GB RAM, with software MATLAB 2017b. In the experiment, we repeated the experiment 20 times and recorded the average results.

### 4.1. Dataset Description

**Figure 2:**
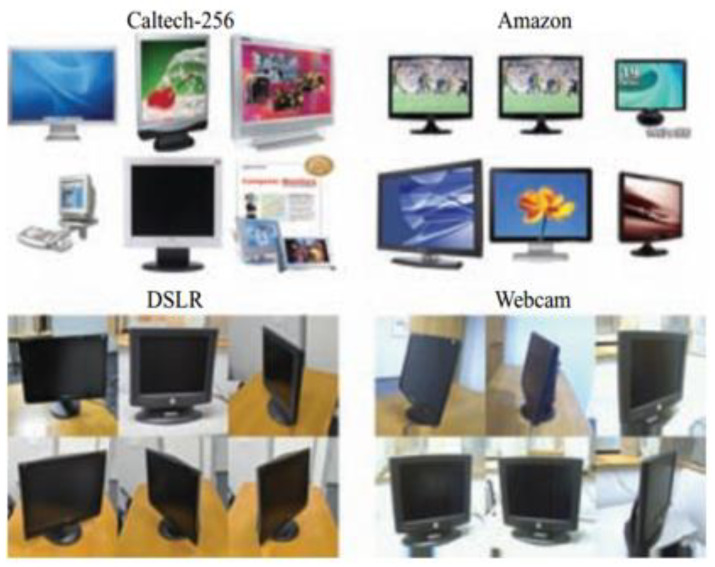
Office + Caltech dataset.

Office + Caltech: It is a visual domain adaptation benchmark dataset, which contains two sub-datasets of Office and Caltech (C), shown in Figure 2. Office contains a total of 4652 pictures with 31 categories from 3 different domain datasets: Amazon (A), DSLR (D), and Webcam (W). In Amazon, each class typically contains an average of 90 images, with an average of 30 images in DSLR or Webcam. Additionally, Caltech is a benchmark dataset for target recognition, containing 30,607 images in 256 categories. In our experiments, we selected 10 shared categories in 4 domains with a total of 2533 images with 800 SURF features. During the experiment, two different domains were selected from the four databases as source and target domain datasets. Thus, we had 12 cross-domain tasks, i.e., C vs. A, C vs. W, C vs. D... and D vs. W.

Office-31: This is a public dataset commonly used to investigate domain adaptation algorithms, which includes three distinct domains: amazon, webcam, and dslr. It contains 4652 images with 31 categories. In our experiments, we carried out a classification task in a 2048-dimensional ResNet-50 feature space and performed six groups of experiments: amazon vs. dslr, amazon vs. webcam, dslr vs. amazon, dslr vs. webcam, webcam vs. amazon, and webcam vs. dslr.

**Figure 3:**
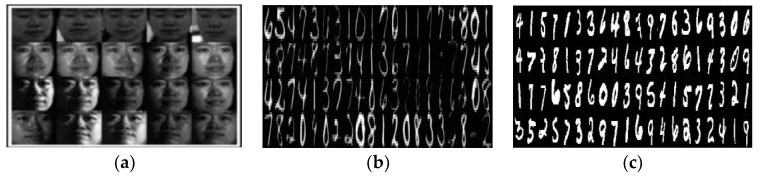
Datasets PIE, USPS, and MNIST. (**a**) PIE; (**b**) USPS; (**c**) MNIST.

PIE: It is a standard facial dataset collected by Carnegie Mellon University, which contains 41,368 face images with a pixel resolution of 32 × 32 from 68 people. These images depict facial postures, lighting, and expressions (as shown in Figure 3a). To verify the performance of the domain adaptation algorithm, we selected five subsets from the dataset: PIE1 (left pose), PIE2 (upward pose), PIE3 (downward pose), PIE4 (frontal pose), and PIE5 (right pose). In each subset (posture), all face images with different expressions and illuminations are changed. In this paper, we selected the source data and the target data, respectively, and constructed 20 cross-domain tasks: PIE1 vs. PIE2, PIE1 vs. PIE3... PIE5 vs. PIE4.

USPS+MNIST: USPS and MNIST are two closely correlated, yet differently distributed, handwritten datasets, sharing 10 numeric categories of 0–9 (as shown in Figure 3b,c). The USPS dataset contains 7291 training samples and 2007 test samples, each with 16 × 16 pixels. There are 60,000 training images and 10,000 test images with 28 × 28 pixels in the MNIST database. During this experiment, 1800 pictures from USPS and 2000 pictures from MNIST were selected to construct source and target domain datasets. All the pictures were converted into grayscale images with 16 × 16 pixels. We constructed two recognition tasks, i.e., USPS as the source domain and MNIST as the target domain (USPS vs. MNIST) and vice versa (MNIST vs. USPS).

### 4.2. Experiment Setting

To evaluate the performance of our algorithm, we compared our approach with several state-of-the-art domain adaptation approaches categorized into three classes: the traditional methods, e.g., 1-NN [48], KELM, and SDA; the shallow feature adaptation methods, e.g., GFK, JDA, STDA-CMC [49], W-JDA, and JDA-CDMAW [50]; and the deep transfer methods, e.g., DAN, DANN, and CAN. In addition, our method is divided into three cases: SCDMDA0 represents SCDMDA+1NN, SCDMDA1 represents SCDMDA+KELM, and SCDMDA2 represents SCDMDA+KTELM. In addition to KELM, SCDMDA1, and SCDMDA2, other algorithms all choose 1-NN as the basic classifier. We applied classification accuracy as the evaluation metric for each algorithm and its formula is as follows:

(19) $$Accuracy=\frac{correctly\_classified\_samples}{total\_samples}\times 100\%$$

To achieve optimal functionality of SCDMDA and KTELM, we set k=100,100,100,512 on Office+Caltech, USPS+MNIST, PIE, and Office-31 datasets, respectively, $T_{\max}=20$, $\alpha \in \left[10^{-2},10^{0}\right]$, $\beta \in \left[10^{-2},10^{1}\right]$, $\lambda_{1}\in \left[10^{-1},10^{3}\right]$, $\theta \in \left[10^{-3},10^{0}\right]$, and $\lambda_{2}\in \left[10^{-2},10^{0}\right]$ for all datasets. For comprehensive comparison, the results of some methods are provided in their original papers or their publicly available codes. We tuned parameters according to their default parameters in released codes or their respective literature.

### 4.3. Results and Analysis

We applied our method, compared algorithms, and performed classification experiments on the Office+Caltech, USPS+MNIST, PIE, and Office-31 datasets. The results are presented in Table 2 and Table 3. From Table 2, conclusions can be obtained as follows:

1. Table 2 summarizes the accuracies of all methods on Office+Caltech, PIE, and USPS+MNIST datasets with shallow feature representation, and the optimal result of each row in the table among all the methods is presented in bold. Our method SCDMDA (0–2) outperforms any other compared methods. The total average accuracy of SCDMDA2 on the 34 tasks is 74.9%, which achieves a 28.75% improvement compared with the baseline SDA. This verifies that CDMA is effective in reducing the between-domain distribution discrepancy and improving the knowledge transfer ability of SDA.
2. SCDMDA2 outperforms SCDMDA0 and SCDMDA1, indicating that KTELM and KELM have higher accuracy than 1-NN, and that the cross-domain mean constraint on the source domain is effective for domain adaptation.
3. Similarly, the semi-supervised feature extraction method SCDMDA0 works better than SWTDA-CMC. An explanation is that CDMA is a better distribution discrepancy measurement criterion than MMD, so our method works quite well in reducing domain bias. SCDMDA0 and SWTDA-CMC are better than JDA, JDA-CDMAW, and W-JDA. This illustrates that the category separability of LDA and the original structure information of graph regularizers are important for the classification task.
4. The accuracy of SDA, GFK, JDA, STDA-CMC, W-JDA, JDA-CDMAW, and SCDMDA (0–2) outperforms 1-NN in most cases, showing the importance of feature extraction in classification tasks. Most domain adaptation techniques such as JDA, STDA-CMC, W-JDA, JDA-CDMAW, and SCDMDA (0–2) achieve higher accuracy than SDA, due to cross-domain shared feature extraction with few or no same distribution samples in the target domain. The accuracy of JDA, STDA-CMC, W-JDA, JDA-CDMAW, and SCDMDA (0–2) generally improves shared feature extraction through joint distribution adaptation.

In addition, results on the Office-31 dataset with ResNet-50 features are shown in Table 3. We observe the classification results obtained by 1-NN, KELM, JDA, STDA-CMC, JDA-CDMAW, and SCDMDA (0–2) and some deep domain adaptation methods such as DAN, DANN, and CAN to verify the effectiveness of our approach on this more complex dataset. SCDMDA2 outperforms other methods on all six groups of domain adaptation tasks. In particular, for the amazon vs. dslr domain adaptation task, it achieves a higher accuracy (91.16%). It further validates that our method, equipped with deep generic features, can further reduce the cross-domain distribution discrepancy and achieve the best adaptation performance, demonstrating the potential of our method.

Table 4 shows the running-time comparisons of SCDMDA (0–2) with 1-NN, KELM, JDA, STDA-CMC, and JDA-CDMAW on the PIE1 vs. PIE2 dataset. The following can be seen: (1) Because JDA, STDA-CMC, JDA-CDMAW, and SCDMDA (0–2) require 20 iterations for label refinement, these approaches consume more time than 1-NN, and KELM. (2) JDA-CDMAW needs less running time than JDA due to no requirement for the construction of the interdomain distribution divergence matrix. (3) SCDMDA (0–2) and STDA-CMC take more time than JDA-CDMAW to obtain the within- and between-class scatter matrices and graph Laplacian matrix (4) The time cost of SCDMDA2 requires more time than SCDMDA1 to compute the cross-domain mean constraint term for domain adaptation. Because KELM is faster than 1-NN, SCDMDA1 needs less time than SCDMDA0. 

### 4.4. Sensitivity Analysis

**Figure 4:**
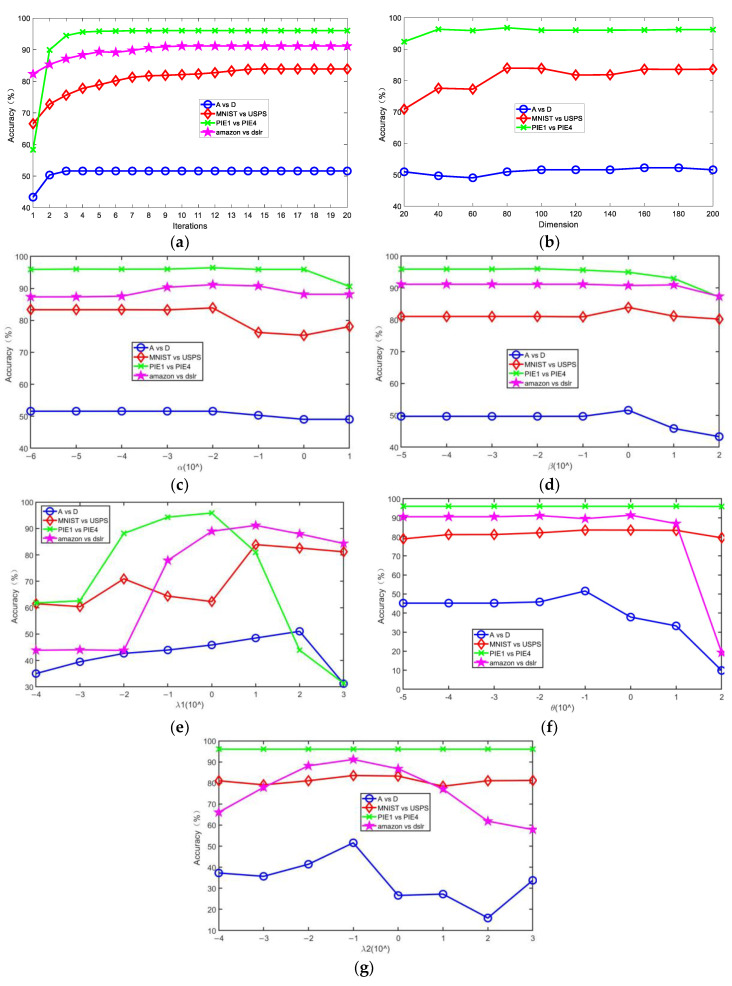
Effect of number of iterations, dimensions of subspace, *α*, *β*, $\lambda_{1}$, *θ*, and $\lambda_{2}$ on accuracy.

In this section, we conducted some experiments to investigate the influence on the accuracy of SCDMDA2, including the number of dimensions of the subspace, the graph tradeoff parameter α, the SDA tradeoff parameter β, and the sparse regularization parameter $\lambda_{1}$ in SCDMDA; and the cross-domain mean constraint control parameter θ and the model regular tradeoff parameter $\lambda_{2}$ of KETLM. In addition, we needed to observe the convergence of the designed algorithm. Then, we carried out experimental analysis on four cross-domain tasks, including A vs. D, MNIST vs. USPS, PIE1 vs. PIE2, and amazon vs. dslr datasets, and the results are shown in Figure 4.

In Figure 4a, we observe that the accuracy of SCDMDA2 grows with the increasing number of iterations on the four sets of datasets and gradually stabilizes after the 8^th^ iteration, verifying its good convergence.

Figure 4b illustrates the experiments with the subspace dimension $k\in \left\{20,40,\cdots ,200\right\}$ on 3 datasets, which indicated the impact of the subspace dimension on our method. It can be observed that SCDMDA2 obtains strong robustness when $k\in \left[80,120\right]$. Moreover, we did not perform this experiment on the amazon vs. dslr datasets, because the Office-31 dataset with ResNet-50 feature is robust with regard to different dimensions for feature transfer learning.

We ran SCDMDA2 with other parameters fixed $\alpha \in [10^{-6},10^{1}]$. From Figure 4c, we can see that the accuracy rises first and then falls with α increasing and obtains optimal performance when $\alpha =10^{-2}$ on 4 datasets. When α is too small, the original structure information cannot be exploited. On the contrary, if it is too large, other terms in Equation (13) will not work and hurt the proposed algorithm. 

Similar situations also occur in Figure 4d–g. The accuracy curve increases first and then decreases with the increase of β, $\lambda_{1}$, θ, and $\lambda_{2}$. When $\beta \in [10^{-2},10^{1}]$, $\lambda_{1}\in [10^{-1},10^{2}]$, 

$\theta \in [10^{-2},10^{0}]$, and $\lambda_{2}\in [10^{-2},10^{-1}]$, SCDMDA2 works well. If β, $\lambda_{1}$, θ, and $\lambda_{2}$ are too small, label information will be lost, the sparsity of the projection matrix fails to be controlled to reach the optimum, the cross-domain mean constraint term does not’ work, and KTELM will decline. In other words, the control of these parameters is beneficial to our approach when the parameter values are reasonable.

**Figure 5:**
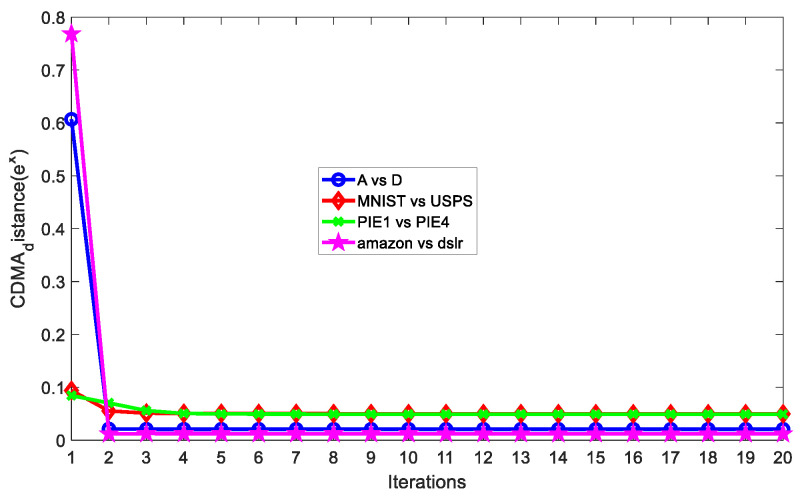
CDMA distances versus number of iterations.

We ran SCDMDA with KTELM on A vs. D, MNIST vs. USPS, PIE1 vs. PIE2, and amazon vs. dslr datasets with the optimal parameter settings, and computed the CDMA distance (shown in Figure 5) according to its definition as follows:

(20) $$\mathrm{CDMA}\_\mathrm{dist}\left(Z_{S},Z_{T}\right)=\left\{\begin{array}{cc} \frac{\sum_{i=1}^{n_{S}+n_{T}}\sqrt{{\left\|z_{i}-\mu \right\|}_{2}^{2}}}{n_{S}+n_{T}} & \;,\;no\;\;label\;\;\;in\;\;target\;domain\\ \frac{\sum_{c=1}^{C}\sqrt{{\left\|z_{S}^{\left(c\right)}-\mu_{T}^{\left(c\right)}\right\|}_{2}^{2}}+\sum_{c=1}^{C}\sqrt{{\left\|z_{T}^{\left(c\right)}-\mu_{S}^{\left(c\right)}\right\|}_{2}^{2}}}{n_{S}+n_{T}} & \;,\;other \end{array}\right.$$

We can see that as the iteration number increases, SCDMDA reduces the between-domain marginal and conditional distribution difference, leading to a smaller CDMA distance and higher accuracy. Therefore, SCDMDA can extract better shared features across domains.

### 4.5. Visualization Analysis

**Figure 6:**
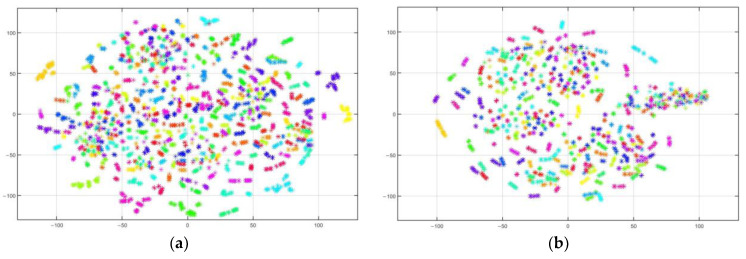

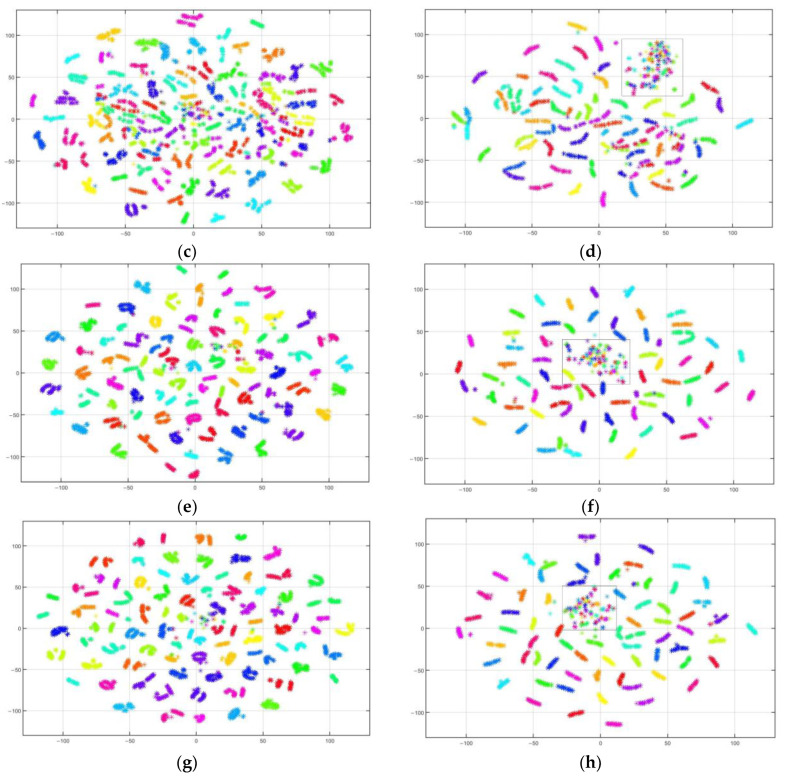
Scatter diagram of feature visualization on PIE1 vs. PIE2. (**a**) Source domain feature. (**b**) Target domain feature. (**c**) Source domain JDA feature. (**d**) Target domain JDA feature. (**e**) Source domain STDA-CMC feature. (**f**) Target domain STDA-CMC feature. (**g**) Source domain SCDMDA feature. (**h**) Target domain SCDMDA feature.

We further display the t-distributed stochastic neighbor embedding (t-SNE) visualization plots of feature embedding for the PIE1 vs. PIE2 classification task. t-SNE, as a dimension-reduction method, transforms high-dimensional data into a low-dimensional space for visualization. In t-SNE visualization plots, clusters of the same color are closer, clusters of different colors are more distant, and the extracted features are more discriminative. The visualized source and target features derived from raw data, JDA, STDA-CMC, and SCDMA were drawn in the feature scatterplot Figure 6a–h, in which each color represents one of the 68 categories.

As we can see from Figure 6a,b, the original data of the source and target domains before adaptation are highly mixed and difficult to distinguish. At the moment, we can easily obtain a poor classifier trained on the source domain. It can be seen from Figure 6c–f that JDA and STDA-CMC can unite samples with the same category and partly separate samples with different categories. Compared with JDA and STDA-CMC from Figure 6d,f, the small number of easily misclassified samples of SCDMDA in the boxes of Figure 6h can be especially observed, showing that SCDMDA can effectively obtain more discriminative and domain-invariant feature representation.

## 5. Conclusions

In this paper, we develop a semi-supervised domain adaptation approach which contains feature-based adaptation and classifier-based adaptation. At the feature-based adaptation stage, we introduce CDMA into SDA to reduce the cross-domain distribution discrepancy and develop SCDMDA for domain-invariant feature extraction. At the classifier-based adaptation stage, we select KELM as the baseline classifier. To further enhance knowledge transfer ability of the proposed approach, we design a cross-domain mean constraint term and add it to KELM to construct KTELM for domain adaptation. Through feature and classifier adaptation, we can learn more discriminatory feature representations and obtain higher accuracy. Comprehensive experiments on several visual cross-domain datasets show that SCDMDA with KTELM significantly outperforms many other state-of-the-art shallow and deep domain adaptation methods.

In the future, we plan to explore the interdomain metric criterion in more depth. At present, CDMA is superior to MMD in reducing cross-domain distribution differences; however, its computational complexity is greater than that of MMD. To solve this problem, better metrics can be developed to improve the distribution differences between domains.

## Figures and Tables

**Table 1:** Description of image datasets.

Dataset	Type	No. of Samples	Feature Dimension	Type Number	Subsets
Office	Object	1410	800	10	A, W, D
Caltech	Object	1123	800	10	C
USPS	Digit	1800	256	10	USPS
MNIST	Digit	2000	256	10	MNIST
Office-31	Image	4652	2048	31	amazon, webcam, dslr
PIE	Face	11,554	1024	68	PIE1,..., PIE5

**Table 2:** Accuracy (%) comparison of different algorithms on Office+Caltech, PIE, and USPS+MNIST datasets.

Dataset/Methods	1-NN	KELM	SDA	GFK	JDA	STDA-CMC	W-JDA	JDA-CDMAW	SCDMDA0	SCDMDA1	SCDMDA2
USPS vs. MNIST	44.70	46.70	27.50	46.45	59.65	63.90	62.35	60.35	66.75	76.80	**76.85**
MNIST vs. USPS	65.94	68.28	62.83	67.22	67.28	79.22	76.11	73.06	76.06	83.44	**83.89**
**Average**	55.32	57.49	45.17	56.84	63.47	71.56	69.23	66.70	71.40	80.12	**80.37**
PIE1 vs. PIE2 (1)	26.09	26.46	27.69	26.15	58.81	72.44	58.87	77.72	**86.86**	84.53	84.53
PIE1 vs. PIE3 (2)	26.59	27.08	28.55	27.27	54.23	73.53	58.15	67.71	81.68	82.66	**82.72**
PIE1 vs. PIE4 (3)	30.67	31.09	41.00	31.15	84.50	93.93	86.19	93.18	95.76	**96.03**	**96.03**
PIE1 vs. PIE5 (4)	16.67	17.89	15.38	17.59	49.75	63.85	56.56	60.11	74.94	76.65	**76.65**
PIE2 vs. PIE1 (5)	24.49	26.86	31.78	25.24	57.62	74.97	63.78	75.30	81.42	84.81	**84.84**
PIE2 vs. PIE3 (6)	46.63	46.63	51.41	47.37	62.93	69.06	64.95	75.31	80.02	80.64	**82.60**
PIE2 vs. PIE4 (7)	54.07	54.46	77.05	54.25	75.82	88.37	80.71	83.84	90.39	93.69	**93.81**
PIE2 vs. PIE5 (8)	26.53	26.96	33.21	27.08	39.89	54.47	40.32	66.54	**72.00**	69.36	69.67
PIE3 vs. PIE1 (9)	21.37	22.09	24.37	21.82	50.96	72.33	59.66	72.39	78.72	81.00	**81.15**
PIE3 vs. PIE2 (10)	41.01	40.95	46.59	43.16	57.95	67.34	61.02	75.63	81.83	82.87	**82.93**
PIE3 vs. PIE4 (11)	46.53	47.73	77.20	46.41	68.45	85.46	79.06	85.10	90.15	93.63	**94.38**
PIE3 vs. PIE5 (12)	26.23	26.84	41.18	26.78	39.95	60.36	48.47	58.03	74.14	**77.02**	**77.70**
PIE4 vs. PIE1 (13)	32.95	34.33	46.49	34.24	80.58	94.24	84.24	91.39	96.13	96.97	**97.03**
PIE4 vs. PIE2 (14)	62.68	62.92	80.91	62.92	82.63	91.04	84.53	90.42	95.40	**96.26**	**96.26**
PIE4 vs. PIE3 (15)	73.22	73.65	86.27	73.35	87.25	91.18	87.50	89.58	93.20	**95.10**	**95.10**
PIE4 vs. PIE5 (16)	37.19	38.17	56.31	37.38	54.66	75.49	59.13	68.93	84.62	84.56	**85.60**
PIE5 vs. PIE1 (17)	18.49	20.23	25.09	20.35	46.46	70.02	52.76	61.85	**76.11**	75.90	75.90
PIE5 vs. PIE2 (18)	24.19	24.80	43.95	24.62	42.05	57.70	43.22	66.36	73.42	**79.56**	79.31
PIE5 vs. PIE3 (19)	28.31	28.98	53.00	28.49	53.31	66.85	55.51	71.26	78.74	**86.58**	**86.58**
PIE5 vs. PIE4 (20)	31.24	33.70	55.69	31.33	57.01	78.64	58.52	77.02	83.90	87.44	**87.47**
**Average**	34.76	35.59	47.16	35.35	60.24	75.06	64.16	75.38	83.47	85.26	**85.51**
C vs. A (1)	23.70	54.49	45.72	41.02	44.78	46.03	48.12	44.68	50.21	60.75	**62.32**
C vs. W (2)	25.76	48.14	35.59	40.68	41.69	42.37	44.07	41.69	46.10	55.25	**57.29**
C vs. D (3)	25.48	43.95	43.31	38.85	45.22	49.68	47.13	45.86	50.96	54.78	**55.41**
A vs. C (4)	26.00	45.06	37.76	40.25	39.36	41.41	41.41	38.56	42.83	48.26	**48.44**
A vs. W (5)	29.83	42.03	38.31	38.98	37.97	39.32	40.00	38.31	43.05	49.15	**52.54**
A vs. D (6)	25.48	44.59	31.21	36.31	39.49	38.22	38.22	43.31	38.85	45.22	**51.59**
W vs. C (7)	19.86	35.71	33.66	30.72	31.17	32.24	31.88	32.32	33.30	37.31	**38.47**
W vs. A (8)	22.96	38.94	31.63	29.75	32.78	33.92	33.09	33.61	36.85	41.02	**41.13**
W vs. D (9)	59.24	76.43	87.26	80.89	89.17	87.90	89.17	89.81	89.81	**92.99**	**92.99**
D vs. C (10)	26.27	34.73	31.88	30.28	31.52	32.15	31.34	30.45	33.57	36.95	**37.49**
D vs. A (11)	28.50	36.01	32.36	32.05	33.09	32.67	32.88	32.67	32.46	**46.03**	**46.03**
D vs. W (12)	63.39	66.44	87.12	75.59	89.49	90.17	91.19	91.19	89.83	**91.86**	**91.86**
**Average**	31.37	47.21	44.65	42.95	46.31	47.17	47.38	46.87	48.99	54.97	**56.30**
**Total Average**	34.77	40.98	46.15	39.29	55.51	65.01	58.53	64.81	70.59	74.27	**74.90**

**Table 3:** Accuracy (%) comparison of different algorithms on Office-31 (Resnet-50).

Datasets/Algorithms	1-NN	KELM	JDA	DAN	DANN	CAN	STDA-CMC	JDA-CDMAW	SCDMDA0	SCDMDA1	SCDMDA2
amazon vs. dslr	79.12	79.12	85.54	78.60	79.70	85.50	82.73	85.54	82.13	90.56	**91.16**
amazon vs. webcam	75.85	75.85	82.52	80.50	82.00	81.50	79.75	82.89	82.64	86.42	**88.55**
dslr vs. amazon	60.17	60.24	66.56	63.60	68.20	65.90	68.09	67.13	69.08	73.52	**73.62**
dslr vs. webcam	95.97	95.97	97.36	97.10	96.90	98.20	98.62	97.36	98.49	**98.99**	**98.99**
webcam vs. amazon	59.92	59.96	68.83	62.80	67.40	63.40	66.31	69.08	68.44	73.73	**73.84**
webcam vs. dslr	99.40	99.40	99.20	99.60	99.10	99.70	**99.80**	99.20	**99.80**	**99.80**	**99.80**
**Average**	78.41	78.42	83.33	80.40	82.20	82.40	82.55	83.53	83.43	87.17	**87.66**

**Table 4:** Consuming time of different approaches on PIE1 vs. PIE 2.

Algorithm	1-NN	KELM	JDA	STDA-CMC	JDA-CDMAW	SCDMDA0	SCDMDA1	SCDMDA2
Time(s)	2.72	2.02	187.26	221.34	96.69	137.60	134.78	173.93

## Data Availability

The datasets generated during and/or analyzed during the current study are available from the corresponding author on reasonable request.
